# Exercise workload: a key determinant of immune health – a narrative review

**DOI:** 10.3389/fimmu.2025.1617261

**Published:** 2025-07-24

**Authors:** Xinxin Shi, Lunuo Hu, David C. Nieman, Fei Li, Peijie Chen, Hui Shi, Yue Shi

**Affiliations:** ^1^ School of Exercise and Health, Shanghai University of Sport, Shanghai, China; ^2^ Human Performance Laboratory, North Carolina Research Campus, Appalachian State University, Kannapolis, NC, United States; ^3^ School of Athletic Performance, Shanghai University of Sport, Shanghai, China; ^4^ Shanghai Key Laboratory of Human Performance, Shanghai University of Sport, Shanghai, China; ^5^ Research Institute for Doping Control, Shanghai University of Sport, Shanghai, China; ^6^ Department of Rheumatology and Immunology, Ruijin Hospital, Shanghai Jiao Tong University School of Medicine, Shanghai, China

**Keywords:** exercise intensity, immune health, moderate-intensity exercise, immune cell function, cytokine regulation

## Abstract

The total exercise workload is an important factor influencing immune health. Appropriately prescribed physical activity can mitigate the detrimental effects of chronic inflammation, bolster the body’s defenses against both infectious and non-infectious diseases, and decelerate the immunosenescence process. Physiological and immune system responses to moderate and strenuous exercise workloads vary markedly. This narrative review summarizes current findings on the impacts of moderate-intensity exercise, high-intensity interval training, and strenuous and prolonged exercise on immune health, elucidating their specific effects and underlying mechanisms. Additionally, the role of challenging environmental conditions in shaping immune responses to exercise is also briefly considered. The insights presented here are intended to guide healthy individuals in selecting evidence-based exercise training protocols that are compatible with both health promotion and immune health. Moreover, this review offers prospective research directions, particularly regarding personalized exercise regimens and the interaction between exercise and environmental factors, providing valuable perspectives for scholars within the field of exercise immunology.

## 1 Introduction

The immune system is a key defensive mechanism that safeguards human health. It identifies and eliminates foreign pathogens, while monitoring and maintaining internal homeostasis. This complex system comprises innate and adaptive immunity, jointly forming the body’s first and second lines of defense against infections and disease (1–3). Modern lifestyle changes, such as prolonged sedentary behavior, irregular sleep patterns, and unhealthy diets have significantly influenced the immune system, resulting in immune dysfunction and heightened disease risk. According to the World Health Organization (WHO), the global incidence of measles reached 10.3 million cases in 2023, representing an almost 60% increase from the previous year. Cancer incidence continues to rise, with approximately 20 million new cases and about 9.7 million deaths worldwide in 2022, and is projected to increase by 77% by 2050 (4). Exercise, as an effective regulatory measure, has been shown to enhance immune cell activity and restore immune system balance, thereby bolstering disease resistance. Consequently, exercise interventions have emerged as an important strategy to confront modern lifestyle challenges, improve immune health, and prevent diseases (5–7).

The immunomodulatory effects of exercise depend on exercise intensity and the total exercise workload (8). Moderate-intensity exercise at recommended workloads (150–300 minutes per week as defined by multiple public health agencies) has proven effective in enhancing immune cell function, strengthening the body’s anti-infection capacity, and improving overall defense. For instance, regular moderate-intensity training can lower the risk of upper respiratory tract infections (URTIs) (9), improve survival rates and reduce inflammatory cytokine levels in mice with acute pneumonia (10), and even mitigate age-related declines in immune function (11). High-intensity interval training (HIIT) at recommended levels has emerged as an effective protocol for improving exercise performance, skeletal muscle capacity (12), cardiovascular health (13), and immune health (14). Strenuous and prolonged exercise may lead to immunosuppression lasting from 3 to 72 hours, during which immune cell counts and functions decline in peripheral blood, increasing susceptibility to infections (8, 15–17). Thus, different exercise workloads exert notably distinct effects on immune health, underscoring the need to select appropriate exercise intensities and workloads to maximize immune health benefits and minimize potential immune impairment.

**Figure 1:**
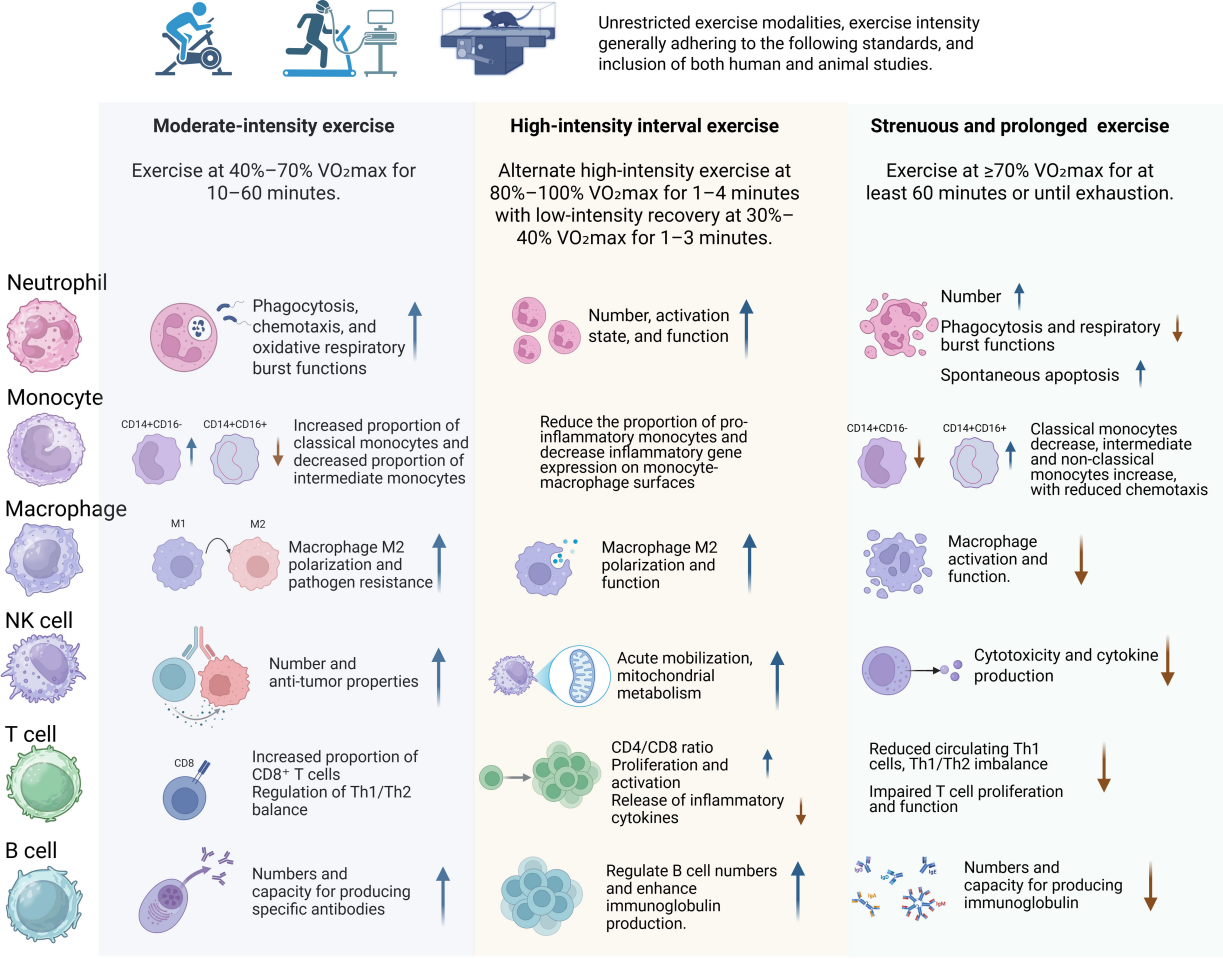
The impact of various exercise workloads on immune cells in both human populations and mice models.

In this context, the present study aims to review the existing literature to analyze the effects of different types of exercise workloads on immune cell function (**Figure 1**), cytokine profiles (e.g., IL–6, TNF–α, IL–10), mucosal immunity, and immunoglobulin production. The goal is to improve scientific understanding regarding the mechanisms underlying the interactions between exercise and immunity, and to explore how optimized exercise interventions can effectively enhance immune function, including innate cellular responses, regulatory cytokines, and antibody production.

To compile relevant literature for this review, we conducted a structured search of the PubMed, Web of Science, and Scopus databases to identify articles published between January 1990 and March 2025. The following Boolean search string was applied:(‘exercise’ OR ‘physical activity’ OR ‘training’) AND (‘immune function’ OR ‘cytokine’ OR ‘interleukin’ OR ‘TNF’ OR ‘IL-6’ OR ‘IL-10’ OR ‘immunoglobulin’ OR ‘NK cells’ OR ‘T lymphocytes’ OR ‘B lymphocytes’ OR ‘neutrophils’ OR ‘macrophages’ OR ‘inflammation’) AND (‘moderate intensity’ OR ‘HIIT’ OR ‘high-intensity interval training’ OR ‘strenuous’ OR ‘prolonged’ OR ‘endurance’ OR ‘resistance’). Inclusion criteria required studies to be published in English, to report original experimental or observational data (human or animal) on exercise-induced changes in immune parameters, and to clearly define the type and intensity of exercise involved. To ensure completeness, additional relevant studies were retrieved by manually reviewing the cited references of core articles identified in the initial search.

## 2 Defining exercise workloads and intensity

According to the American College of Sports Medicine (18), supported by international guidelines (19, 20), exercise intensity is commonly categorized as follows: low intensity is defined as <40% VO_2max_, moderate intensity ranges from 40% to 70% VO_2max_, and high intensity typically spans from 60% to 84% VO_2max_.

Low-intensity exercise is generally suitable for beginners and individuals in recovery, as it does not induce significant fatigue. Common activities include leisurely walking and slow cycling. Moderate-intensity exercise is appropriate for most healthy adults and helps improve cardiovascular function and aerobic capacity (21). High-intensity exercise, on the other hand, demands a certain level of physical fitness. During such exercise, individuals experience rapid breathing and typically struggle to maintain a conversation, but high-intensity training can significantly improve VO_2max_ and enhance performance. High-intensity interval training (HIIT), characterized by alternating between brief bursts of near-maximal effort and recovery periods, is a key example of high-intensity exercise that elicits significant physiological responses. Research indicates that, compared to sustained moderate-intensity exercise, HIIT can significantly improve VO_2max_ in a shorter duration (22). However, other studies suggest that intense and prolonged high-intensity exercise may temporarily suppress the immune system, increasing the risk of infection, particularly in competitive environments (17). Therefore, understanding the differences in immune responses to various exercise workloads is crucial for designing training programs that optimize immune health and disease prevention.

## 3 The impact of different exercise workloads and intensities on the immune system

### 3.1 Effects of moderate-intensity exercise on the immune system

Acute and chronic moderate-intensity exercise training is generally regarded as an immune system adjuvant that has multiple beneficial effects in stimulating both innate and adaptive immunity and lowering the risk for infectious diseases (17).

#### 3.1.1 Effects on neutrophils

Multiple studies have revealed positive effects of regular moderate-intensity exercise on neutrophil function. Such exercise enhances neutrophil phagocytic activity and oxidative burst capacity, significantly improving their function (23). In healthy women, moderate-intensity exercise improves neutrophil chemotaxis, phagocytosis, and bactericidal capacity more distinctly than high-intensity exercise (24). In overweight adults with cardiovascular risk factors, moderate-intensity training helps maintain neutrophil bactericidal activity (25). Moreover, moderate-intensity exercise increases total glutathione (GSH) levels, elevates antioxidant reserves, enhances mitochondrial membrane potential, and delays neutrophil apoptosis (26).

Research by Mooren et al. showed that the combined effect of fasting and moderate-intensity exercise significantly enhances oxidative burst activity in neutrophils post-exercise (27). Hypoxic conditions can further augment exercise-induced immune effects, increasing neutrophil phagocytosis of E. coli and activating NADPH oxidase, thereby enhancing oxidative burst and pathogen clearance (28). The duration of moderate-intensity exercise is also important; acute exercise (30 minutes of moderate-intensity walking) can increase circulating neutrophils in patients with chronic kidney disease (CKD), boosting their anti-infective potential, whereas long-term regular exercise (30 minutes/day, 5 times a week for 6 months) may not significantly influence neutrophil degranulation (29). Furthermore, acute exercise significantly upregulated the gene expression of several cytokines in skeletal muscle, particularly the monocyte chemoattractant CCL2 (MCP-1) and the neutrophil-attracting CXCL2 (Gro-β), suggesting that skeletal muscle may attract immune cells through the secretion of these signaling molecules to promote inflammation and tissue repair post-exercise (30). Collectively, moderate-intensity exercise exerts multifaceted positive effects on neutrophils, improving their phagocytic and bactericidal functions, antioxidant defenses, and environment-dependent immune responses, ultimately strengthening the body’s immune defenses.

#### 3.1.2 Effects on monocyte-macrophage populations

Moderate-intensity exercise training can affect the distribution of monocyte subsets and macrophage function. After 10 weeks of moderate-intensity exercise in healthy adults, the proportion of classical monocytes (CD14^++^/CD16^-^) in peripheral blood increased, while intermediate monocytes (CD14^+^/CD16^+^) and non-classical monocytes (CD14^-^/CD16^++^) decreased. Additionally, the expression of CD16, TLR2, and TLR4 on monocyte surfaces was reduced, weakening the pro-inflammatory phenotype of monocytes (31), indicating that sustained moderate exercise promotes immune regulation.

Long-term moderate-intensity exercise induces metabolic rewiring in macrophages, enhancing mitochondrial function and shifting macrophage responses toward a more anti-inflammatory state by modulating biosynthetic and bioenergetic processes (32). Studies have also shown that exercise training can regulate the immune microenvironment, promoting the polarization of macrophages toward the M2 phenotype (33). For example, a study by Knudsen et al. demonstrated that endurance training induces systemic increases in IL-13, which acts via the IL-13Rα1–Stat3 axis to reprogram skeletal muscle metabolism. This signaling promotes mitochondrial biogenesis and fatty acid oxidation, and indirectly facilitates a shift toward an anti-inflammatory M2 macrophage phenotype (34).

It promotes a shift toward M2-related gene expression and reduces M1-related gene expression, driving macrophages toward an M2 phenotype and lowering inflammation. For example, after 8 weeks of moderate-intensity exercise, sedentary women showed an increase in M2 markers (such as Dectin-1 and IL-10) and a decrease in M1 markers (such as MCP-1) on monocytes, thereby influencing macrophage polarization (35). Blanks et al. further demonstrated that habitual exercisers displayed lower CCR2 expression on intermediate monocytes and a greater propensity for M2 macrophage polarization following moderate-intensity cycling (36). These effects were partially sex-dependent, as shown by Blanks et al. (37), who linked post-exercise CCR2 downregulation to ERK1/2 signaling specifically in female participants (37). Moreover, moderate exercise can lower the expression of CXCR2 on CD14^+^/CD16^+^ monocytes in the peripheral blood of obese adults, reducing inflammatory monocyte infiltration into tissues (such as adipose tissue) and alleviating obesity-related chronic low-grade inflammation (38).

Although long-term endurance training and the late recovery phase after acute bouts tend to promote M2-like macrophage polarization, the early phase following acute exercise is dominated by the mobilization of pro-inflammatory monocytes and an increase in M1-like macrophage infiltration, particularly in skeletal muscle tissues (39–41). Voskoboynik et al. also indicated in their study that long-term training favors the M2 phenotype to promote recovery and metabolic remodeling, while acute exercise initiates M1 activation to respond to immune stress (42). This biphasic response underscores the need to consider temporal dynamics when evaluating immune adaptations to exercise. In addition, acute aerobic exercise induces robust macrophage infiltration and chemokine (e.g., CXCL12) release in skeletal muscle, especially in individuals with type 2 diabetes. The altered immune profile highlights the role of exerkines and cross-talk between myocytes and immune cells in tissue adaptation (43).

While above human trials animal studies provide insights into the underlying mechanisms, similar effects on macrophage function have been observed in animal studies. In mouse models, moderate-intensity training improves macrophage function and enhances disease resistance. Regular moderate-intensity training can reverse age-related declines in antiviral characteristics of peritoneal and alveolar macrophages (11). Pre-exercise training for 6 days strengthens macrophage resistance to HSV-1 in mice (44), and 8 weeks of moderate exercise increases alveolar and interstitial macrophage counts in mice with acute pneumonia, altering expression of alveolar macrophage markers CD64 and MHC II, thereby creating an anti-inflammatory healing environment (10). Furthermore, in aging contexts, exercise exerts systemic immunoregulatory effects across multiple tissues. A recent study in elderly mice found that aerobic training reduces inflammation-related pathways (e.g., NF-κB, TNF) and restores communication between stem cells and immune populations in muscle, brain, and blood. These changes reestablished regenerative niches and reinforced anti-inflammatory macrophage phenotypes, indicating a rejuvenation of the immune microenvironment (45).

These findings highlight the key role of moderate-intensity exercise in improving macrophage distribution, function, and phenotype, thus enhancing immune defense and reducing chronic inflammation.

#### 3.1.3 Effects on natural killer cells

Moderate-intensity exercise mainly enhances NK cell number, activity, and antitumor properties, thereby improving immune surveillance and tumor defense. Acute moderate-intensity exercise transiently increases NK cell counts, which return to baseline within a few hours (46). NK cells, integral to innate immunity, play a pivotal role in tumor surveillance (47, 48). Chronic moderate-intensity exercise has demonstrated a mobilizing effect on NK cells, hence augmenting their activity and anti-tumor capabilities (49). Gannon et al. found that moderate-intensity cycling significantly increased peripheral NK cell counts and NK cytotoxic activity (NKCA) (50). Six weeks of moderate-intensity exercise also increased the cytotoxic NK cell subset in whole blood, implying enhanced antitumor potential (51).

In addition to their roles in cytotoxic defense against viruses and tumors, NK cells are also critically involved in the clearance of senescent cells, a process that contributes to tissue homeostasis and aging regulation. Recent human studies show that even short durations of moderate-intensity exercise (e.g., 15 minutes at ~55% heart rate reserve) can mobilize cytotoxic NK cells into the bloodstream (52). These effects extend to functionally relevant subsets, such as CD56^dim^ and CD57^+^ NK cells, which have been observed to increase after moderate exercise in pediatric hematopoietic stem cell transplant recipients (53) and cancer patients undergoing chemotherapy (54). Moreover, muscle-derived cytokines like IL-15 appear to promote NK cell proliferation and cytotoxicity, including against senescent targets (55). Supporting this, a recent meta-analysis demonstrated that senescent NK cell subsets (e.g., CD57^+^) remain elevated for more than an hour post-exercise, reinforcing the hypothesis that physical activity may contribute to immune-mediated senolysis (56).

These findings further support the concept that moderate-intensity exercise not only enhances immune surveillance of NK cells in the context of tumor defense but may also contribute to the clearance of senescent cells, thus playing a role in aging and tissue homeostasis.

#### 3.1.4 Effects on T lymphocytes

Moderate-intensity exercise can alter T-cell subset distribution and enhance specific T-cell subset functions. Acute moderate exercise significantly increases CD8^+^ T-cell counts in lymphoma patients, with levels returning to baseline within 30 minutes post-exercise (57). Regular moderate-intensity training prevents declines in CD4^+^ T-cell counts observed after strenuous exercise (58) and enhances CD4^+^ T-cell proliferation and cytokine production following vaccination, improving vaccine efficacy (59). CD4^+^ and CD8^+^ T cells can differentiate into Th1 or Th2 subsets, with Th1 cells producing pro-inflammatory cytokines (IL-2, IFN-γ) for cell-mediated immunity and Th2 cells producing anti-inflammatory cytokines (IL-4, IL-10) for humoral immunity (60). Moderate-intensity training improves both Th1 and Th2 functions, maintaining Th1/Th2 balance. Studies show that acute moderate exercise increases the IL-2/IL-4 ratio in allergic rhinitis patients, reducing inflammation (61), while 2 months of moderate training in healthy males significantly increased IFN-γ and IL-2 levels in PBMC cultures, enhancing Th1 responses (62). In older adults, regular moderate-intensity exercise increases CD28 expression and Th1 cell count, improving overall immunity (63). Furthermore, Drela et al. observed that moderate exercise enhances IL-2 production, mitigating immunosenescence in older individuals (64).

Animal models confirm that moderate-intensity exercise can adjust Th1/Th2 balance, boost IFN-γ production, reduce IL-4 and IL-10, and favor Th1-mediated responses to intracellular pathogens. For example, 8 weeks of moderate exercise significantly raised the IFN-γ/IL-4 ratio in mice, enhancing Th1 responses (65). Kohut et al. found that 8 weeks of moderate-intensity training in aged mice augmented Th1 functions and cytokine release, improving infection resistance (66). In influenza-infected mice, moderate-intensity training reduced pulmonary IFN-γ gene expression and shifted a Th1 inflammatory to a Th2 anti-inflammatory response, increasing survival rates (67).

Overall, moderate-intensity exercise supports T-cell function and sustains Th1/Th2 equilibrium, promoting a balanced immune response that enhances both cell-mediated immunity (Th1) and humoral immunity (Th2). This shift is essential for maintaining immune function, enhancing immune defense, and promoting healthy aging by preventing excessive inflammatory responses (68, 69).

#### 3.1.5 Effects on B lymphocytes

Moderate-intensity exercise positively influences B-cell function and antibody production, strengthening immune defense. Acute moderate-intensity training increased total immunoglobulin E (IgE) levels in allergic rhinitis patients (61) and enhanced immunoglobulin M (IgM) and immunoglobulin G (IgG) levels following influenza vaccination in older adults (70), improving vaccine responses. In obese postmenopausal women, 15 weeks of moderate-intensity training significantly elevated serum IgG, IgM, and IgA (71), confirming its beneficial effects on immunoglobulin production.

Regular moderate-intensity exercise also increases salivary secretory IgA (SIgA) in older adults (72, 73), bolstering mucosal immunity as a first line of defense. In animal models, prolonged moderate-intensity training reduced B-cell numbers in the duodenal lamina propria but significantly raised IgA levels, improving resistance to gut pathogens (74). In older mice, moderate exercise increased B-cell and IgA^+^ B-cell counts and factors related to IgA synthesis, thus strengthening intestinal IgA production and immune function (75). Hence, moderate-intensity exercise fosters B-cell proliferation and antibody generation, enhancing mucosal immunity and pathogen defense.

#### 3.1.6 Effects on cytokines

Moderate-intensity exercise exerts a wide range of effects on cytokines, especially in the modulation of critical cytokines like IL-6, TNF-α, and IL-10.The release of IL-6 from contracting skeletal muscle, first identified as a ‘myokine’ nearly two decades ago, represents a hallmark cytokine response to endurance exercise. IL-6 levels can rise up to 100-fold during prolonged activity and play key roles in hepatic glucose output, adipose tissue lipolysis, and anti-inflammatory signaling (76, 77). Moderate-intensity exercise has a dual effect on IL-6, encompassing both short-term immune activation and long-term anti-inflammatory adaptations. Acute moderate-intensity exercise typically triggers a significant increase in IL-6 release due to skeletal muscle contractions, marking a short-term immune response. This increase in IL-6 usually returns to baseline levels within 24 hours (78). Additionally, IL-6 acts not only as a pro-inflammatory mediator during this initial response but also helps modulate anti-inflammatory factors such as IL-10, maintaining immune homeostasis after exercise (79). This mechanism is essential for regulating post-exercise immune function. In contrast, long-term moderate-intensity exercise produces distinct effects. Sustained moderate exercise helps regulate IL-6 levels, promoting immune balance, alleviating chronic low-grade inflammation, and improving metabolic health (80). For example, 6 weeks of regular training reduced the IL-6/IL-10 ratio in patients with CKD (29). One year of moderate-intensity combined with resistance training (MICT+RT) significantly lowered plasma IL-6 in type 2 diabetes patients (81). Animal studies similarly show that moderate exercise lowers IL-6 in septic and diabetic rats, mitigating inflammation and improving survival (82, 83).

Moderate exercise also lowers TNF-α levels, reducing inflammation. Acute exercise decreases TNF-α production by monocytes, mediated by the sympathetic nervous system and β2-adrenergic receptors (84). Prolonged moderate training can maintain long-term suppression of TNF-α, diminishing chronic inflammation. For instance, 6 months of training lowered TNF-α mRNA expression in fluorosis-exposed mice (85). In humans, moderate exercise reduces TNF-α in individuals with high-fat diets or obesity, decreasing low-grade chronic inflammation (86, 87). Moreover, moderate exercise enhances IL-10 secretion, fostering an anti-inflammatory environment. In patients with chronic fatigue syndrome, older adults, and those with systemic lupus erythematosus, moderate-intensity exercise significantly increases IL-10 levels and reduces inflammation (88–90). Animal models corroborate this effect, showing that moderate exercise increases IL-10 concentration and improves immune function while reducing inflammation (74, 86, 91).

Although moderate-intensity exercise has been widely studied for its acute immunological effects, it is important to note that not all individuals can safely or consistently perform at this level. For such populations, low-intensity physical activity may offer an accessible and physiologically beneficial alternative.

While low-intensity physical activity (e.g., walking, stretching, tai chi, Pilates) may not trigger the acute and measurable immune shifts observed with higher-intensity modalities, emerging research suggests it confers significant long-term health benefits. Studies have shown that low-intensity exercise avoids post-exercise immunosuppression (92), promotes anti-inflammatory cytokine expression such as IL-4 (93), enhances dendritic cell and T cell proliferation (94), and elevates mucosal sIgA levels in older adults (95). Furthermore, programs such as low-impact Pilates have been found to alter adaptive immune cell distributions (96), while regular participation may help mitigate age-related immune decline (97). These findings underscore the potential of low-intensity physical activity as a safe and sustainable immunoregulatory strategy, particularly for vulnerable populations.

### 3.2 Effects of high-intensity interval training on the immune system

The number of HIIT studies investigating immune responses in human participants is limited but expanding. Most studies indicate that immune responses to HIIT at recommended levels are rapid but modest and transient, similar to the beneficial responses to moderate, sustained exercise bouts.

#### 3.2.1 Effects on neutrophils

HIIT has a positive role in increasing neutrophil counts, enhancing their functionality, activating innate immune responses, and bolstering pathogen defense. Acute HIIT significantly increases neutrophil counts (98) and elastase release upon lipopolysaccharide (LPS) stimulation (99), indicating greater neutrophil activation and function. Compared to continuous moderate exercise, HIIT activates neutrophil-mediated innate immune responses earlier (100). Moreover, HIIT improves neutrophil phagocytic and respiratory burst capacities (31), chemotaxis, and mitochondrial function (101). Bartlett et al. found that HIIT improves neutrophil function in rheumatoid arthritis patients, including migration accuracy, enhanced phagocytosis, and increased reactive oxygen species (ROS) production (14). HIIT also reduces excessive neutrophil extracellular trap formation (NETosis) in older men, potentially preventing inflammatory diseases (102).

Animal studies show that HIIT enhances phagocytic capacity in type 1 diabetic rats (103) and reduces lung neutrophil infiltration in acute lung injury models by lowering bronchoalveolar lavage fluid (BALF) MPO levels, thereby alleviating inflammatory damage (104). These findings underscore the positive effects of HIIT on neutrophil function and innate immunity.

#### 3.2.2 Effects on monocyte-macrophage populations

Acute High-Intensity Interval Training (HIIT) triggers a temporary immune activation in monocytes. Studies show that a single session of HIIT significantly decreases TLR2 expression on classical and CD16^+^ monocytes, indicating its anti-inflammatory properties and the ability to modulate monocyte immune responses (105). Additionally, acute HIIT also indirectly influences monocyte activation by decreasing postprandial triglyceride levels, increasing CD11b and reducing CD36 expression on monocytes (106).

Chronic HIIT exerts more pronounced effects on monocytes and macrophages, especially in individuals with obesity and metabolic disorders. De Matos et al. demonstrated that 8 weeks of regular HIIT reduced the proportion of non-classical monocytes (CD14^-^/CD16^++^) and intermediate monocytes (CD14^+^/CD16^+^) in obese adults, restoring the balance of monocyte subsets (107). Moreover, 12 weeks of HIIT in obese men with non-alcoholic fatty liver disease (NAFLD) altered monocyte subset distribution and activation states, decreasing inflammatory gene expression (TLR4, CD11b, and CD14), thereby alleviating inflammation (108). Animal studies further support that long-term HIIT can reduce pro-inflammatory monocyte subsets in the liver and stimulate anti-inflammatory responses, markedly improving the inflammatory condition of non-alcoholic steatohepatitis (NASH). This effect was more pronounced compared to long-term moderate-intensity training (109). These findings suggest that HIIT is effective in ameliorating obesity-related immune dysfunction and inflammation.

Regarding macrophage polarization, long-term HIIT reduces M1 markers (CD86) while increasing M2 markers (CD206), which helps mitigate liver inflammation (110). Animal studies also show that regular HIIT can modulate the activation of monocyte-macrophages in muscle tissue, reducing M1 macrophages and increasing M2 macrophages (111), while also reversing high-fat diet-induced M1 polarization and further promoting M2 polarization, thereby improving metabolic and immune status (112).

In summary, acute HIIT triggers short-term immune activation in monocytes, while chronic HIIT offers lasting anti-inflammatory benefits by balancing monocyte subsets and promoting macrophage polarization towards the M2 phenotype, especially in individuals with obesity and metabolic disorders.

#### 3.2.3 Effects on natural killer cells

Acute HIIT rapidly mobilizes natural killer (NK) cells into the peripheral blood, particularly cytotoxic subpopulations expressing CD57, CD158a, NKG2D, TIM-3, and CXCR3 (cNK), which return to baseline levels after one hour, thereby balancing the distribution of NK cell subsets (54). Furthermore, both animal and human studies confirm that prolonged HIIT training increases circulating NK cell numbers (113).

Additionally, both acute and long-term HIIT have been shown to enhance NK cell activity and function. A single session of HIIT boosts NK cell activity by elevating the activities of antioxidant enzymes and their biological roles (98). Lin et al. demonstrated that 6 weeks of HIIT enhances NK cell mitochondrial membrane potential, reduces mitochondrial oxidative burden (MOB), and increases perforin and granzyme B levels, thereby improving NK cell cytotoxicity against tumor cells (51). In cancer patients, HIIT has been shown to increase NK cell counts and tumor infiltration, augmenting antitumor immunity (114, 115), while higher training doses yield better tumor NK cell infiltration (115). Thus, HIIT supports NK cell mobilization, activity, mitochondrial function, and antitumor cytotoxicity, strengthening immune surveillance.

#### 3.2.4 Effects on T lymphocytes

Acute HIIT has a significant impact on the quantity and function of T cells. Studies show that acute HIIT increases the number of CD4^+^ and CD8^+^ T cells in peripheral blood and raises the CD4/CD8 ratio, but this increase typically returns to near pre-exercise levels within 3 hours post-exercise (99). Additionally, acute HIIT rapidly boosts the number of circulating regulatory T cells (Tregs), exerting a short-term immunoregulatory effect. Krüger et al. (116) found that acute HIIT significantly increased Treg numbers in peripheral blood immediately after exercise and up to 3 hours post-exercise, indicating that HIIT can quickly enhance immune regulation through Treg modulation (116). However, acute HIIT may also induce redox imbalance in T cells, leading to reduced T cell proliferation in response to superantigen (SEB) stimulation, although this does not affect early activation or apoptosis (117).

The effects of long-term HIIT on T cells are more persistent, particularly in individuals with low cardiorespiratory fitness (CRF). Dorneles et al. found that long-term HIIT significantly increased the proportion of CD4^+^CD25^high^CD127^low^ Tregs and CD39^+^ memory Tregs in obese men with low CRF, enhancing Treg immunoregulatory function through upregulation of CD39 and CD73 (118). Furthermore, after 3 weeks of HIIT training, the CD4/CD8 T cell ratio in peripheral blood was significantly increased compared to moderate-intensity training, suggesting that HIIT may be more effective than sustained moderate-intensity exercise in regulating immune cell ratios and reducing inflammation (111). Câmara et al.observed that after 5 weeks of HIIT, IL-4 (Th2 cytokine) levels decreased, while the IFN-γ/IL-4 ratio significantly increased, indicating a shift towards Th1 cells, which enhanced CD4^+^ T cell autophagy and anti-apoptotic capacity, mitigating hypoxia-induced damage (119). Although long-term HIIT improves T cell function, it may exacerbate immune aging in certain populations, such as older women at high risk for breast cancer, leading to a decrease in CD4^+^ T cell counts and a lower CD4/CD8 ratio, suggesting that HIIT may promote immune aging (120).

In conclusion, acute HIIT exerts a rapid immunoregulatory effect by increasing Tregs and modulating T cell subsets. Long-term HIIT improves T cell subset distribution and enhances immune regulation. However, the effects of HIIT may vary across different populations, particularly in terms of immune aging, and long-term HIIT may need to be tailored to individual conditions.

#### 3.2.5 Effects on B lymphocytes

HIIT acutely alters B-cell counts, leading to a transient decrease immediately post-exercise, followed by a return to baseline within 30 minutes (117). HIIT also increases immunoglobulin production, enhancing mucosal immunity. A single HIIT session significantly increased salivary sIgA levels especially in female athletes (121). Three weeks of HIIT elevated sIgA secretion rates in healthy males, maintaining mucosal immunity (122). Correspondingly, in obese women, 8 weeks of HIIT raised serum IgA, improving mucosal defense (123). These studies collectively suggest that acute HIIT boosts mucosal immunity temporarily by increasing sIgA, while chronic HIIT can lead to more sustained improvements in serum IgA and immune defense.

#### 3.2.6 Effects on cytokines

The effects of high-intensity interval training on cytokines are characterized by a combination of acute responses and prolonged adaptive modifications. Acutely, HIIT elevates IL-6, TNF-α, and IL-10, reflecting an initial immune activation. However, the magnitude of increase in plasma cytokine levels following recommended levels of HIIT is modest, especially when compared to responses following prolonged, strenuous running or cycling. Healthy individuals and patients with colorectal cancer, chronic heart failure, or type 1 diabetes show significant but moderate increases in IL-6 and TNF-α after a single HIIT session, returning to baseline within several hours to a day (124–129). IL-10 also rises acutely, initiating anti-inflammatory processes (130, 131). Long-term HIIT training reduces chronic low-grade inflammation similar to the effects of long-term moderate aerobic exercise training regimens. Twelve weeks of HIIT lower IL-6 and TNF-α in obese adolescents and type 2 diabetes patients (132, 133), while enhancing IL-10 secretion to support anti-inflammatory capacity (134, 135). Animal studies similarly confirm that 8 weeks of HIIT reduce IL-6 and TNF-α in kidney-injured mice, improving inflammatory status (136, 137).

Furthermore, chronic HIIT training diminishes acute immune fluctuations and modulates cytokine expression in various disease models, promoting health. In obesity, type 2 diabetes, and Parkinson’s disease, HIIT effectively lowers TNF-α and alleviates chronic inflammation (138–140). It also consistently elevates IL-10, enhancing anti-inflammatory capacity in obese men, type 2 diabetes patients, and post-percutaneous coronary intervention (PCI) patients (135, 141, 142). Basic research further supports IL-10 enhancement by HIIT, mitigating age-related inflammation and improving immunity (139, 143) (144). Collectively, HIIT training can effectively enhance immune function and modulate inflammation status by regulating levels of IL-6, TNF-α, and IL-10, thereby having significant benefits for health and disease management.

### 3.3 Effects of prolonged and strenuous exercise on the immune system

Prolonged and intensive exercise induces physiological and metabolic stress and a corresponding and profound effect on both innate and adaptive immunity.

#### 3.3.1 Effects on neutrophils

Strenuous or exhaustive exercise elicits pronounced innate immune responses. After exhaustive exercise, neutrophil numbers significantly increase (neutrophilia), particularly after prolonged (over 2 hours) activity (145). This surge is transient but accompanies declines in phagocytic and oxidative burst functions (146). For instance, during militarized training, the number of neutrophils in healthy men increased significantly and sustained at elevated levels (147). Marathon running increases peripheral band neutrophils, indicating bone marrow release (148). Furthermore, in animal experiments, it was observed that elevated neutrophil infiltration into muscle, kidney, and liver may exacerbate tissue injury (149, 150), and neutrophil depletion can be a factor alleviating liver damage following exhaustive exercise (150).

Acute strenuous exercise also impairs neutrophil phagocytic function and increases ROS levels (151), causing oxidative stress, reducing mitochondrial membrane potential, and accelerating neutrophil apoptosis (26). In elite male athletes, neutrophil phagocytic function significantly declined 2 hours post-exercise and persisted at 6 and 24 hours (152). Furthermore, high-intensity exercise to fatigue affects degranulation responses, which return to normal within 24 hours, whereas persistent functional suppression occurs after prolonged low-intensity exercise to exhaustion (153). Such findings illustrate the complexity of strenuous exercise’s impact on neutrophils, potentially contributing to post-exercise immunosuppression.

#### 3.3.2 Effects on monocyte-macrophage populations

Acute and long-term intense/exhaustive exercise affects the monocyte-macrophage system through different mechanisms, activating pro-inflammatory responses, disrupting immune function, and causing tissue damage, thereby increasing susceptibility to infections. Studies have found that after a single bout of exhaustive exercise in healthy subjects, the number of classical monocytes (CD14^++^/CD16^-^) decreases, while the numbers of intermediate (CD14^+^/CD16^+^) and non-classical monocytes (CD14-/CD16^++^) increase. Additionally, LPS stimulation leads to reduced IL-6 and IL-10 production by monocytes, while TNF-α secretion increases, indicating a shift to a pro-inflammatory state (154). Acute intense exercise also increases the expression of TLR2 and TLR4 on monocyte surfaces and decreases HLA-DR expression, weakening the antigen-presenting ability of monocytes (155). Animal studies show that after acute exhaustive exercise, M1 macrophages in the liver are activated (156), and macrophages migrate to muscle, liver, and kidney, promoting the release of pro-inflammatory cytokines and exacerbating tissue damage (157–159). Moreover, acute exhaustive exercise reduces macrophage survival and antiviral capacity (160). These studies suggest that acute intense exercise activates the monocyte-macrophage system, amplifying the inflammatory response and increasing tissue damage. Chronic intense exercise primarily affects monocytes by weakening their chemotaxis and altering immune function. Studies have shown that three weeks of intense exercise training may impair monocyte chemotaxis (161). Furthermore, chronic intense exercise can impair macrophage microbial killing mechanisms, increasing infection susceptibility (162).

#### 3.3.3 Effects on natural killer cells

Strenuous exercise significantly affects NK cell counts and activity. During acute strenuous exercise, NK cell counts rise, possibly due to catecholamine-mediated demargination (163, 164). However, after over one hour of intense activity, NK cell counts drop below baseline levels and may remain low for up to 8 hours, returning to resting levels after 24 hours (152, 165). While NK cell activity may initially surge, it eventually becomes suppressed after exercise, prolonging the immunosuppression window and increasing infection risk (16, 166). Studies have confirmed that after acute intense exercise, NK cell function is suppressed, with a decrease in IFN-γ production upon *in vitro* stimulation, and this suppressive effect persists for up to 2 hours post-exercise (163). Eda et al. found that exercising at 75% V̇O_2max_ for 60 minutes significantly reduced NK cell activity in healthy men, with the suppression persisting until the following day (167). Kohut’s study also showed that exhaustive exercise reduces NK cell cytotoxicity in mice (168). Overall, the decrease in NK cell activity after intense exercise is a key factor in temporary immune suppression.

However, prolonged high-intensity exercise has less impact on NK cell numbers but also leads to NK cell functional suppression, particularly in cases of overtraining. This functional exhaustion results in a reduced immune response, increasing the risk of infections.

#### 3.3.4 Effects on T lymphocytes

Strenuous exercise induces complex effects on T cells, including temporary changes in T-cell numbers, subset shifts, functional adjustments, and altered cytokine production. After acute high-intensity or prolonged exercise, both CD4^+^ and CD8^+^ T cell counts in the blood increase, with a more significant rise in CD8^+^ T cells. During the recovery phase (30 minutes to 6 hours post-exercise), CD8^+^ T cell counts rapidly decline, possibly even below baseline levels, leading to lymphocytopenia. Within 24 hours, CD8^+^ T cell counts generally return to resting levels. Meanwhile, Th1 cytokine production (e.g., IFN-γ) decreases, while Th2 cytokines (e.g., IL-10) increase, shifting the immune response toward Th2, which may impair antiviral and antitumor immunity (60, 68). Studies by Lancaster et al. found that, after acute intense exercise, the number of Th1 cells significantly decreased, and their ability to produce IFN-γ was notably reduced, while Th2 cell numbers and IL-4 production remained unchanged (69, 169). Kakanis et al.confirmed that after strenuous cycling, Th2 cytokines increased in peripheral blood, while Th1 cytokine production increased at 4 and 8 hours post-exercise, showing a transient shift from Th1 to Th2 (170). Mouse studies also indicated that after acute intense exercise to exhaustion, Th1 cytokines (e.g., IFN-γ) decreased, while Th2 cytokines (e.g., IL-4) either remained unchanged or increased, potentially weakening immunity against intracellular pathogens and raising infection risk (168). Acute high-intensity exercise also increases Tregs and FoxP3^+^ Tregs in the peripheral blood of adolescent swimmers (171), suggesting immune regulation may change post-exercise. However, long-term high-intensity training has been shown to enhance Treg-mediated immune suppression, which may reduce excessive immune responses but also increase infection risk (60).

Furthermore, after prolonged intense training, peripheral blood CD8^+^ T cell numbers decrease, and the CD4/CD8 ratio is downregulated. CD8^+^ T cells may also exhibit aging phenotypes (e.g., CD57^+^ KLRG1^+^), accompanied by a Th1/Th2 shift towards Th2, weakening immune responses and increasing the risk of infections (60, 68). For instance, a 12-year longitudinal study on Olympic athletes found that long-term high-intensity training could lead to abnormal T cell characteristics, with a Th2-dominant immune response (172).

In addition to these shifts in T-cell subsets, regulatory T cells (Tregs) have emerged as central mediators of skeletal muscle adaptation to exercise. Langston et al. showed that both acute and chronic endurance exercise rapidly increase the accumulation of CD4^+^Foxp3^+^ Tregs in skeletal muscle, where they suppress IFN-γ–mediated mitochondrial damage and preserve oxidative metabolism. Functional Treg depletion impaired muscle bioenergetics and reduced endurance performance, confirming their critical role in maintaining tissue integrity during exercise adaptation (173). This dual function of Tregs—modulating inflammation and supporting metabolic homeostasis—has also been highlighted in a systematic review by Proschinger et al. (174). The review found that chronic aerobic or resistance training elevates circulating Tregs and enhances their suppressive capacity (e.g., IL-10, TGF-β secretion), while acute exercise responses vary depending on sampling time, intensity, and phenotypic markers used. Furthermore, Becker et al. identified IL-6Rα signaling as essential for Treg maturation during exercise. Mice lacking IL-6Rα in T cells showed impaired muscle regeneration and reduced Treg activity following training. These findings suggest that muscle–immune communication during exercise requires both cytokine signaling and Treg coordination to ensure proper immune function and tissue repair (175).

These studies underscore the importance of Tregs in both regulating the immune response and supporting metabolic adaptation in response to exercise. Tregs contribute to the preservation of muscle integrity and mitochondrial function, playing an essential role in the body’s long-term adaptation to physical activity.

#### 3.3.5 Effects on B lymphocytes

Both acute and chronic intense exercise affect B-cell function. Acute exercise typically leads to a decrease in immunoglobulin levels, while prolonged intense exercise can induce an immune suppression window, impairing immune defense and increasing infection risk. Specifically, acute prolonged strenuous exercise lowers B-cell numbers (147), compromises antibody production (146, 165), and affects delayed-type hypersensitivity (176). For example, 5–7 days of intensive military training reduced serum IgG, IgA, and IgM in healthy men (147). Chronic intense exercise, such as training in elite athletes, is often associated with significant reductions in IgG, IgA, and IgM, particularly after high-intensity, prolonged endurance training, leading to temporary immune suppression (177). This immune suppression primarily affects B-cell function and reduces sIgA levels, weakening mucosal immunity and increasing infection risk (178). In male cyclists, IgA and IgM levels significantly dropped post-exercise, correlating with higher respiratory infection susceptibility (179). Rugby and elite swimming training likewise lowered sIgA levels and secretion rates, raising URTIs incidence (180, 181). These findings indicate that intense exercise can impair B-cell function and mucosal immunity, increasing infection risk.

#### 3.3.6 Effects on cytokines

The effects of strenuous exercise on cytokines mirror the acute immune response that is triggered by exercise. Strenuous exercise triggers a rapid and large increase in IL-6 and TNF-α levels post-exercise, which can last for around 24 hours, particularly after extreme events like ultramarathons and triathlons where IL-6 and TNF-α levels significantly surpass baseline values (182, 183). Exhaustive exercise also increases cytokine expression in the liver, heart, skeletal muscle, and intestines, exacerbating inflammation and tissue damage (157, 184–187). Nutritional interventions including carbohydrate supplementation can modulate IL-6 changes, helping regulate inflammation and recovery (188). However, chronic strenuous exercise may intensify inflammation, as TNF-α levels rise with repeated bouts of intense activity (65, 189). IL-10 responses vary after strenuous exercise. Some studies show that excessive exercise boosts IL-10 as a counter-regulatory mechanism (190, 191), while others find reduced IL-10 production in certain athletes (192). Notably, the ratio of IL-10 to TNF-α varies across different tissues, exhibiting an anti-inflammatory impact in muscle while adipose tissue tends to be pro-inflammatory (193). These studies suggest that the effects of strenuous exercise on cytokines exhibit temporal and tissue-specific characteristics, with significant implications for the regulation of inflammatory responses.

Although strenuous exercise is linked to transient immunosuppression, the clinical and performance effects in athletic groups are debated (6). De Araújo et al. found that both long-term moderate-training and intensive-training lifestyles in elderly men were associated with stronger antibody responses to influenza vaccination compared to sedentary peers (194). Experimental studies also indicate that strenuous swimming after rabies vaccination does not reduce antibody titers or survival rates (195). More research is needed to better understand the impact of long-term prolonged and intensive training on immune health, but thus far there is little indication that endurance athletes experience higher rates of infectious disease or immune-related disorders. However, a consistent finding is that URTI rates are higher in endurance athletes during periods that include intensified training and competitive events (196).

### 3.4 Comparative immune signatures across exercise intensities

Immune responses to exercise follow intensity-specific trajectories across multiple effector systems. Moderate-intensity exercise generally promotes immune surveillance and homeostasis, characterized by enhanced neutrophil phagocytosis, M2-skewed macrophage polarization, increased NK cytotoxic activity, improved T cell proliferation, and elevated IL-10 production. These effects are associated with reduced chronic inflammation and improved resistance to infection. In contrast, high-intensity interval training (HIIT) generates acute immune activation, increasing neutrophil, monocyte, and NK cell mobilization, as well as short-term inflammatory markers such as IL-6 and TNF-α. However, long-term HIIT may enhance immunoregulatory capacity depending on baseline fitness.

Strenuous or prolonged exercise, especially when unaccustomed or sustained, tends to cause transient immune dysfunction, including neutrophil degranulation, NK cell suppression, and Th1/Th2 imbalance. This can lead to prolonged inflammatory cytokine elevation, heightening infection susceptibility. Hence, while high-intensity and strenuous exercise have specific adaptive advantages, they must be carefully controlled to avoid immunosuppressive effects.

### 3.5 Environmental modifiers of exercise-induced immune responses

In addition to internal workload characteristics, recent studies have highlighted the importance of extrinsic environmental conditions—such as air pollution, heat, and urban climate factors—in modulating exercise-induced immune responses. These environmental exposures can independently or synergistically affect inflammation, oxidative stress, and mucosal immunity during and after physical activity.

For instance, combined exposure to fine particulate matter (PM_2.5_), ozone (O_3_), and nitrogen dioxide (NO_2_) during endurance exercise has been shown to significantly enhance both airway and systemic inflammation, elevate circulating neutrophil levels, and impair mucosal immune protection (197, 198). Likewise, exercising in high-heat environments (≥35 °C) can amplify the acute release of IL–6 and TNF–α, while delaying immunoregulatory cytokine responses such as IL–10 (199). Heat stress and oxidative damage can compromise epithelial integrity, increase permeability, and lead to immune dysfunction (200, 201). In urban settings, wildfire smoke can compound inflammatory responses and lengthen the immune suppression window (202, 203).

These findings emphasize the necessity of integrating environmental exposure into the design of exercise programs, especially for populations with pre-existing inflammatory conditions or respiratory sensitivity.

## 4 Conclusions and future perspectives

This review underscores the multifaceted effects of varying exercise workloads on the immune system. In summary, moderate-intensity exercise consistently provides the most balanced immune benefits, promoting immune surveillance, improving cellular functions, and reducing chronic inflammation. In contrast, HIIT offers beneficial acute immune activation, enhancing NK cell cytotoxicity and modulating inflammatory markers, though its long-term effects on immune regulation are still under investigation. Prolonged or strenuous exercise, while capable of temporarily boosting immune cell mobilization, is often linked to post-exercise immunosuppression and increased infection risk due to cytokine imbalances. These findings suggest that moderate-intensity exercise is optimal for sustaining immune health, especially for those at risk of metabolic disorders or immune decline.

Future research should focus on inter-individual differences in immune responses under various exercise intensities and explore the interplay between exercise type, individual characteristics (e.g., age, health status, genetic endowment), and immune adaptation. Additionally, understanding how exercise interventions can be optimized to enhance immune function in specific disease states (e.g., cancer, diabetes) remains a crucial research avenue. Moreover, future studies are needed into the effects of exercise on immune cell subpopulations, immune markers, and their functions, as well as investigate the potential mechanisms of diverse exercise modalities and personalized exercise regimens on immune health. Prospective clinical trials and mechanistic studies will help clarify the roles of varying exercise intensities in long-term immune adaptation, ultimately providing valuable evidence-based guidelines for sports medicine and exercise nutrition.

## 5 Limitations

This review has several limitations that should be acknowledged. First, the studies included span a broad range of exercise modalities, populations, and outcome measures, which introduces substantial heterogeneity and limits direct comparability. Second, while moderate- and high-intensity exercise were well-represented, data on low-intensity physical activity remain sparse and often lack robust immunological endpoints. Finally, due to space limitations, certain immunological markers, such as dendritic cell function or immunoglobulin production, were not extensively discussed. These limitations highlight the need for standardized protocols, expanded biomarker profiling, and longitudinal human trials in future research.
